# DNA Barcoding and Comparative Nutritional Profiling of “Cod” (鳕) Products from Chinese E-Commerce Platforms

**DOI:** 10.3390/foods15193575

**Published:** 2026-10-08

**Authors:** Shuai Che, Rong Huang, Chaoqun Wang, Ang Li, Shufang Liu, Zhimeng Zhuang

**Affiliations:** State Key Laboratory of Mariculture Biobreeding and Sustainable Goods, Yellow Sea Fisheries Research Institute, Chinese Academy of Fishery Sciences, Qingdao 266071, China; cheshuai@ysfri.ac.cn (S.C.); 18146670829@163.com (R.H.); wangchaoqun9712@163.com (C.W.); liang@ysfri.ac.cn (A.L.); zhuangzm@ysfri.ac.cn (Z.Z.)

**Keywords:** seafood authentication, DNA barcoding, Gadiformes, nutritional quality, Chinese e-commerce

## Abstract

The commercial designation “cod” (鳕) is applied to multiple fish species, raising concerns regarding seafood authenticity and nutritional consistency. This study combined DNA barcoding with comparative nutritional profiling to authenticate frozen products marketed as “cod” (鳕) from major Chinese e-commerce platforms and to evaluate nutritional differences among the identified species. Among the 36 products analyzed, eight fish species were identified: three Gadiformes species (55.6%) and five non-Gadiformes species (44.4%). Interspecific differences were observed in proximate composition, amino acid composition, free amino acid profiles, and fatty acid composition. True cod species exhibited higher protein contents (>19.0%), lower lipid contents (0.51–1.02%), and higher proportions of EPA + DHA (35.61–37.10% of total fatty acids) than non-Gadiformes species. In contrast, non-Gadiformes species generally contained higher lipid levels (15.02–23.09%), lower proportions of long-chain ω-3 PUFA, and distinct flavor-related amino acid profiles. *Lepidocybium flavobrunneum* showed the lowest EPA + DHA proportion (5.81%) and the highest concentration of bitter-tasting free amino acids (0.661 g/100 g). These findings reveal substantial nutritional heterogeneity among the authenticated species marketed as “cod” (鳕), demonstrating that DNA barcoding provides an essential foundation for linking species identity to nutritional quality assessment.

## 1. Introduction

“Cod” (鳕) is one of the most commercially valuable groups of whitefish traded worldwide, appreciated for its high protein content, low lipid content, mild flavor, and desirable texture [1]. According to the Food and Agriculture Organization (FAO), the global trade value of cod-related products reached approximately USD 14.88 billion in 2024 [2]. Owing to the favorable nutritional and sensory characteristics, cod is widely marketed in various processed forms like fillets, steaks, and surimi, making it an important commodity in the international seafood trade [3]. In the strict biological sense, “cod” refers to fish species within the order Gadiformes, primarily the families Gadidae (e.g., *Gadus* spp.) and Merlucciidae (hakes). Species of the genus *Gadus*, such as Atlantic cod (*Gadus morhua*), Pacific cod (*G. macrocephalus*), and Alaska pollock (*G. chalcogrammus*), are globally recognized as the archetypal “true cods” [4]. In commercial practice, however, “cod” (鳕) is frequently used not as a strict biological concept but as a commercial designation, whose definition varies considerably among countries and regulatory systems. For instance, the U.S. Food and Drug Administration (FDA) Seafood List permits the use of “cod” as a market name for 15 distinct species [5], whereas the Chinese national standard (GB/T 41545-2022) provides recommended guidance for aquatic product labeling but does not establish mandatory species-specific commercial names for “cod” (鳕) [6]. Consequently, different fish species may be marketed under the same commercial name, while identical species may be sold under different names in different regions. Such inconsistencies have increased the complexity of seafood labeling and created challenges for accurate species identification and product traceability.

In recent years, China has become one of the world’s largest importers of cod-related products, with frozen products accounting for a substantial proportion of retail sales [2]. Meanwhile, the rapid expansion of e-commerce has reshaped the retail landscape for aquatic products in China, establishing online platforms as a major consumption channel for seafood [7]. Unlike offline markets, e-commerce platforms present products through webpages featuring photographs and text-based descriptions, preventing consumers from directly examining the physical characteristics of the products [8]. This challenge is particularly pronounced for processed products such as skinless fillets, in which diagnostic morphological characteristics are absent [4]. Previous studies have demonstrated that commercially available “cod” (鳕) comprises multiple species, including other Gadiformes such as the giant grenadier (*Albatrossia pectoralis*, family Macrouridae), as well as taxonomically unrelated species. Notable examples include sablefish (*Anoplopoma fimbria*, family Anoplopomatidae, order Scorpaeniformes), often marketed as “black cod”, and Patagonian toothfish (*Dissostichus eleginoides*, family Nototheniidae, order Perciformes), commonly sold as “Chilean sea bass” or “white cod” [9,10]. More concerning is the inclusion of species such as escolar (*Lepidocybium flavobrunneum*) and oilfish (*Ruvettus pretiosus*, family Gempylidae, order Perciformes), which contain high levels of wax esters associated with gastrointestinal discomfort [11]. Therefore, when consumers rely solely on the label “cod” (鳕) in their purchasing decisions, the actual species of origin often remains ambiguous.

DNA barcoding, particularly based on a ~650 bp fragment of the mitochondrial cytochrome c oxidase subunit I (*COI*) gene, has emerged as one of the most widely applied molecular tools for seafood authentication because of its high accuracy in species identification, even in processed products lacking morphological characteristics [12,13,14]. The U.S. FDA has approved DNA barcoding for species identification to prevent seafood label fraud since 2011 [15,16]. Since its introduction into seafood authentication, DNA barcoding has been extensively applied to detect species substitution and labeling inconsistencies in fishery products, including cod-related products [17,18,19,20]. These studies have substantially improved our understanding of seafood authenticity; however, they have primarily focused on species identification, whereas comparatively little attention has been paid to the nutritional characteristics of authenticated species. Fish species marketed as “cod” (鳕) can differ substantially in lipid content, fatty acid composition, amino acid profiles, and other nutritional attributes [21,22,23]. Consequently, species substitution may alter the nutritional attributes of products marketed under the same commercial designation and influence consumers’ dietary expectations.

To address the dual challenges of species ambiguity and nutritional uncertainty in the Chinese e-commerce “cod” (鳕) market, the present study combined DNA barcoding with comparative nutritional profiling to investigate frozen products marketed as “cod” purchased from two major Chinese e-commerce platforms. First, DNA barcoding was used to definitively identify the species composition of 36 “cod” (鳕) products. Second, a comprehensive comparative analysis of proximate composition, amino acid (hydrolyzed and free) profiles, and fatty acid composition was conducted on the authenticated species. By integrating molecular authentication with nutritional evaluation, this study provides baseline information on species diversity and nutritional variability among commercially marketed “cod” (鳕) products and contributes to improving seafood authenticity assessment and nutritional quality evaluation in the Chinese e-commerce seafood market.

## 2. Materials and Methods

### 2.1. Sample Collection and Preparation

A total of 36 individual frozen products marketed as “cod” (鳕) were purchased from two major Chinese e-commerce platforms, Taobao and Jingdong, between September and November 2025. Products were selected to represent different vendors, commercial labels, countries of origin, and price ranges in order to reflect the diversity of products available to Chinese consumers.

All products were received in frozen condition and immediately stored at −20 °C until further processing. For sample preparation, each product was first thawed overnight at 4 °C. Approximately 30 mg of dorsal white muscle was then aseptically excised from each product and preserved in 95% ethanol for DNA analysis. The remaining edible muscle tissue was subsequently frozen in liquid nitrogen, homogenized using a laboratory grinder (JXFSTPRP-CLN-24, Jingxin Co., Ltd., Shanghai, China), and stored at −80 °C until nutritional analyses.

### 2.2. DNA Barcoding and Species Identification

Total genomic DNA was extracted from ethanol-preserved tissue using a Marine Animal Tissue DNA Kit (Tiangen Biotech, Beijing, China) according to the manufacturer’s instructions. DNA concentration and purity were determined using a NanoDrop One spectrophotometer (Thermo Fisher Scientific, Waltham, MA, USA). The standard 658 bp fragment of the mitochondrial *COI* gene was amplified using universal fish primers FishF1 (5′-TCAACCAACCACAAAGACATTGGCAC-3′) and FishR1 (5′-TAGACTTCTGGGTGGCCAAAGAATCA-3′) [24]. PCR amplification was performed in a 25 μL reaction mixture containing 1 μL genomic DNA (100 ng/μL), 1 μL of each primer (10 μM), 2.5 μL 10× PCR Master Mix, and 19.5 μL nuclease-free water. Amplification conditions followed Ward et al. [24]. PCR products were examined by agarose gel electrophoresis, purified using a commercial PCR purification kit (Watson BioTechnologies Inc., Shanghai, China), and subjected to bidirectional Sanger sequencing by the Beijing Genomics Institute (Qingdao, China).

Sequences were edited and aligned using DNASTAR 5.01 software (DNASTAR, Madison, WI, USA). Final sequences were compared against the GenBank nucleotide database using BLASTn (https://blast.ncbi.nlm.nih.gov/Blast.cgi, accessed on 15 January 2026). Species identity was assigned when sequence similarity was ≥99% together with complete query coverage and concordant taxonomic assignment among the top BLAST matches. A Neighbor-Joining (NJ) phylogenetic tree was constructed in MEGA11 (Molecular Evolutionary Genetics Analysis, version 11; Kumar Lab, Temple University, Philadelphia, PA, USA) based on genetic distances calculated using the best-selected model, Kimura two-parameter (K2P), with 1000 bootstrap replicates. The K2P model and NJ algorithm were selected to facilitate comparison with previous DNA barcoding studies employing the standard *COI* barcode region. Reference sequences representing all identified species were retrieved from GenBank and included in the phylogenetic analysis (Supplementary Table S1) [25,26,27,28,29,30,31,32,33,34,35,36,37]. All *COI* sequences generated in this study were deposited in GenBank and accession numbers are listed in Table S2.

### 2.3. Nutritional Composition Analysis

For species that were identified in multiple commercial products, the muscle tissues from all authenticated samples of the same species were pooled and homogenized together for nutritional analysis. All analytical determinations were carried out in triplicate (technical replicates), and the mean values ± standard deviation (SD) were calculated for subsequent statistical comparisons. Moisture, crude protein (Kjeldahl method, nitrogen conversion factor = 6.25), crude fat (acid hydrolysis), and ash contents were determined according to respective Chinese National Standards [38,39,40,41]. Hydrolyzed amino acids were determined following GB 5009.124-2016 using acid hydrolysis and ion-exchange chromatography [42]. Free amino acids were extracted with sulfosalicylic acid, derivatized, and quantified by high-performance liquid chromatography (HPLC, Agilent 1260, Agilent Technologies, Inc., Santa Clara, CA, USA) according to the method described by Chen et al. [43]. Fatty acid methyl esters (FAMEs) were prepared by acid-catalyzed hydrolysis and transesterification following GB 5009.168-2016, Method III [44]. Fatty acid composition was analyzed using gas chromatography equipped with a flame ionization detector (GC-FID) (Agilent 8890, Agilent Technologies, Inc., Santa Clara, CA, USA). Individual fatty acids were identified by comparison with standard FAME mixtures and quantified using the area normalization method. The results therefore describe the relative composition of the lipid fraction and are expressed as percentages of total fatty acids. All chemicals and reagents were purchased from Sigma-Aldrich (St. Louis, MO, USA).

### 2.4. Evaluation of Protein Quality

Protein nutritional quality was evaluated based on the digestible indispensable amino acid reference ratio (DIAARR), digestible indispensable amino acid score (DIAAS), chemical score (CS) and essential amino acid index (EAAI). The DIAARR and DIAAS were calculated according to the FAO/WHO (2013) amino acid scoring pattern for adults [45], whereas the CS was calculated using whole egg protein as the reference protein. EAAI was calculated according to Shi et al. [46]. Because amino-acid-specific and species-specific true ileal digestibility data were not available for the eight authenticated species, a single overall protein digestibility value of 82% reported for cod muscle [47] was applied uniformly to all amino acids and all species. The resulting values are therefore reported as estimated DIAARR (eDIAARR) and estimated DIAAS (eDIAAS). The essential amino acid with the lowest eDIAAS or CS value was regarded as the first limiting amino acid. The calculation formulas are presented below:

$$\mathrm{eDIAARR}=\frac{C_{A}\times \mathrm{Digestibility}}{C_{\mathrm{FA}0/\mathrm{WHO}}}$$

$$\begin{array}{c} \;\mathrm{eDIAAS}\%=100\times \mathrm{lowest}\;\mathrm{value}\;\mathrm{of}\;\mathrm{eDIAARR} \end{array}$$

$$\begin{array}{c} \;\mathrm{CS}=\frac{C_{A}}{C_{\mathrm{Egg}}} \end{array}$$

$$\begin{array}{c} \;\mathrm{EAAI}=\sqrt[{n}]{\frac{100A}{\mathrm{AE}}\times \frac{100B}{\mathrm{BE}}\times \frac{100C}{\mathrm{CE}}\ldots \times \frac{100H}{\mathrm{HE}}} \end{array}$$

where *C_A_* represents the content of the dietary indispensable amino acid in 1 g of the sample protein (mg/g protein); *C_FAO/WHO_* represents the corresponding dietary indispensable amino acid in 1 g of the reference protein (mg/g protein); *C_Egg_* represents the amino acid content of whole egg protein; *n* represents the number of essential amino acids included in the calculation; *A*, *B*, *C*…*H* are the contents of essential amino acids in the muscle protein (mg/g protein); and *AE*, *BE*…*HE* are the contents of the corresponding amino acids in whole egg protein (mg/g protein).

### 2.5. Statistical Analysis

Statistical analyses were performed using IBM SPSS Statistics 25.0 (IBM Corp., Armonk, NY, USA). Results are presented as mean ± standard deviation (SD). Because the number of commercial products differed among authenticated species, triplicate measurements are technical replicates of pooled species samples.

## 3. Results and Discussion

### 3.1. DNA Barcoding Reveals High Species Diversity and Commercial Name Inconsistency in “Cod” (鳕) Products

**Figure 1:**
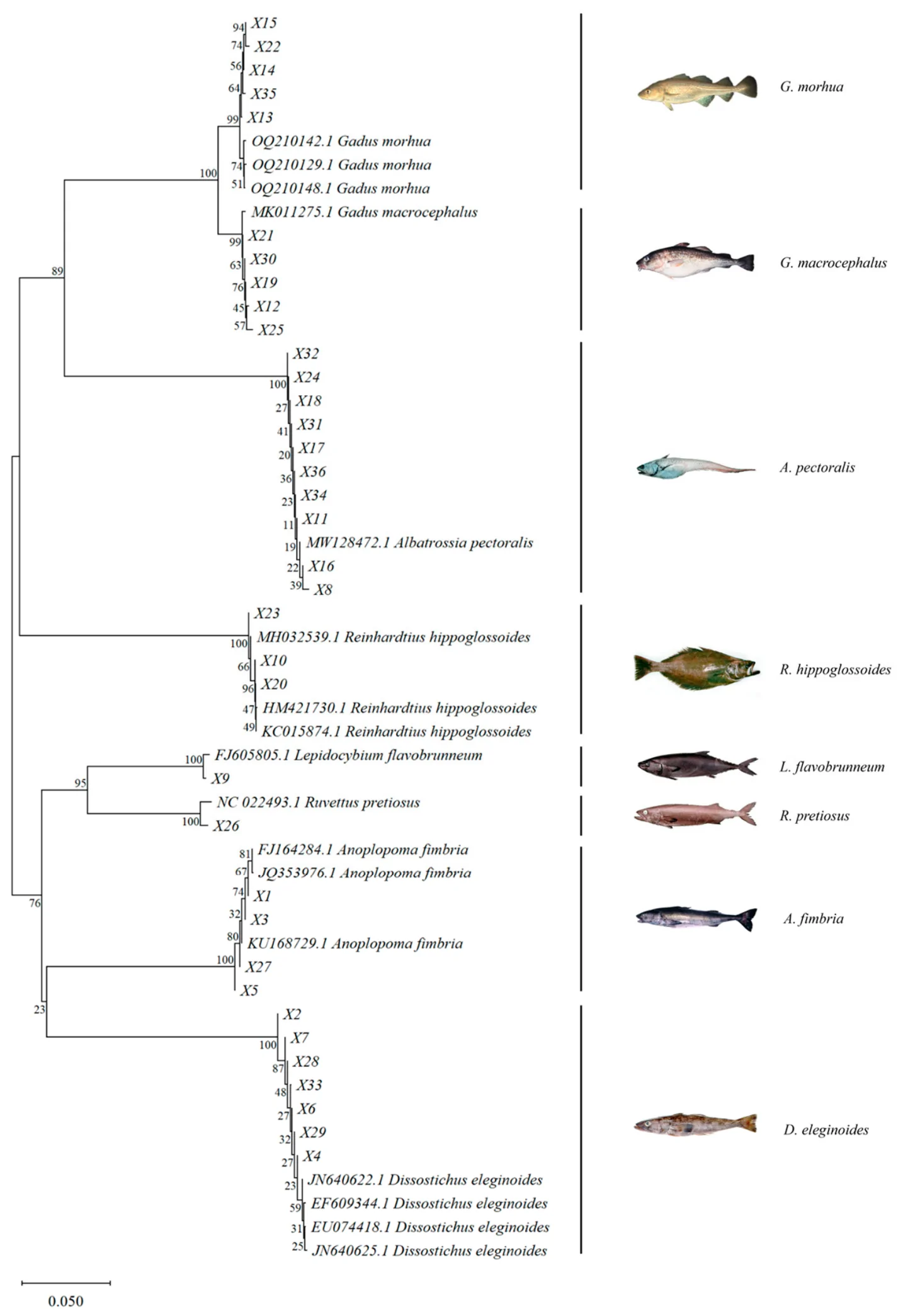
Neighbor-Joining (NJ) phylogenetic tree based on the *COI* gene. Photo credit: FishBase database (https://fishbase.mnhn.fr/search.php, accessed on 18 January 2026).

Mitochondrial *COI* gene sequences (~658 bp) were successfully amplified and sequenced from all 36 frozen products labeled as “cod” (鳕). BLAST analysis against the GenBank database demonstrated that all sequences exhibited ≥99% identity with reference sequences, enabling reliable species-level identification [35]. Intraspecific genetic distances, calculated using the K2P model, ranged from 0.00 to 0.01 (mean = 0.004), while interspecific distances ranged from 0.131 to 0.265 (mean = 0.223), consistent with established DNA barcoding criteria for species discrimination [48]. The NJ phylogenetic tree further supported these results, with all samples clustering into eight well-supported monophyletic clades corresponding to the identified species (bootstrap > 95%) (Figure 1).

DNA barcoding identified eight fish species among the 36 commercial products (Table 1). Only 20 samples (55.6%) belonged to the order Gadiformes, comprising Atlantic cod (*Gadus morhua* Linnaeus, 1758; n = 5) and Pacific cod (*Gadus macrocephalus* Tilesius, 1810; n = 5) (family Gadidae), and giant grenadier (*Albatrossia pectoralis* Gilbert, 1892; n = 10) (family Macrouridae). The remaining 16 products (44.4%) were assigned to five non-Gadiformes species from four orders, including sablefish (*Anoplopoma fimbria* Pallas, 1814; order Scorpaeniformes: Anoplopomatidae; n = 4), Greenland halibut (*Reinhardtius hippoglossoides* Walbaum, 1792; order Pleuronectiformes: Pleuronectidae; n = 3), Patagonian toothfish (*Dissostichus eleginoides* Smitt, 1898; Nototheniidae, n = 7), and a single specimen each of escolar (*Lepidocybium flavobrunneum* Smith, 1843; n = 1) and oilfish (*Ruvettus pretiosus* Cocco, 1833; Gempylidae; n = 1).

Molecular identification results revealed that 44.4% (16/36) of products were not Gadiformes, whereas 55.6% (20/36) belonged to Gadiformes (Table 1), which were comparable to or slightly lower than the name-inconsistent frequencies reported for other processed seafood in Chinese retail markets [49,50], suggesting that the use of the same commercial name for products with different scientific names remains a common issue in commercially processed aquatic products. Notably, eleven products labeled as “Silver Cod” comprised four sablefish and seven Patagonian toothfish, neither of which taxonomically belongs to the family Gadidae. Among the sixteen generically labeled “Cod” (鳕) products, ten were identified as giant grenadier, three were identified as Greenland halibut and three were identified as Pacific cod. All products labeled with explicit scientific or standardized species names (e.g., “Pacific cod”, “Atlantic cod”) were correctly identified, suggesting that products with precise species declarations are more reliable. In contrast, two products labeled only as “White cod” and “Cod pieces” were identified as *L. flavobrunneum* (escolar) and *R. pretiosus* (oilfish), respectively. These two species are of particular concern because of their high wax ester content and potential to cause gastrointestinal discomfort; their sale under a generic “cod” name may mislead consumers who expect a true cod product [11,51].

These results demonstrated that products marketed under the commercial designation “cod” (鳕) encompass a taxonomically diverse group of fishes rather than exclusively representing true cod species (*Gadus* spp.), reflecting the broad commercial use of this name in the Chinese e-commerce market. Similar situations have been reported in several countries, where commercial names are legally permitted to encompass multiple species with distinct taxonomic origins [8,19,20]. Although such naming practices facilitate seafood marketing, they also increase the difficulty of species traceability, particularly for frozen fillets and other highly processed products. In the e-commerce context, consumers rely exclusively on webpage descriptions and cannot physically inspect products prior to purchase [8]. This constraint underscores the importance of accurate species-level information obtained through molecular methods such as DNA barcoding in online seafood retail.

### 3.2. Proximate Composition Reveals Distinct Nutritional Differences Among Authenticated “Cod” (鳕) Species

**Figure 2:**
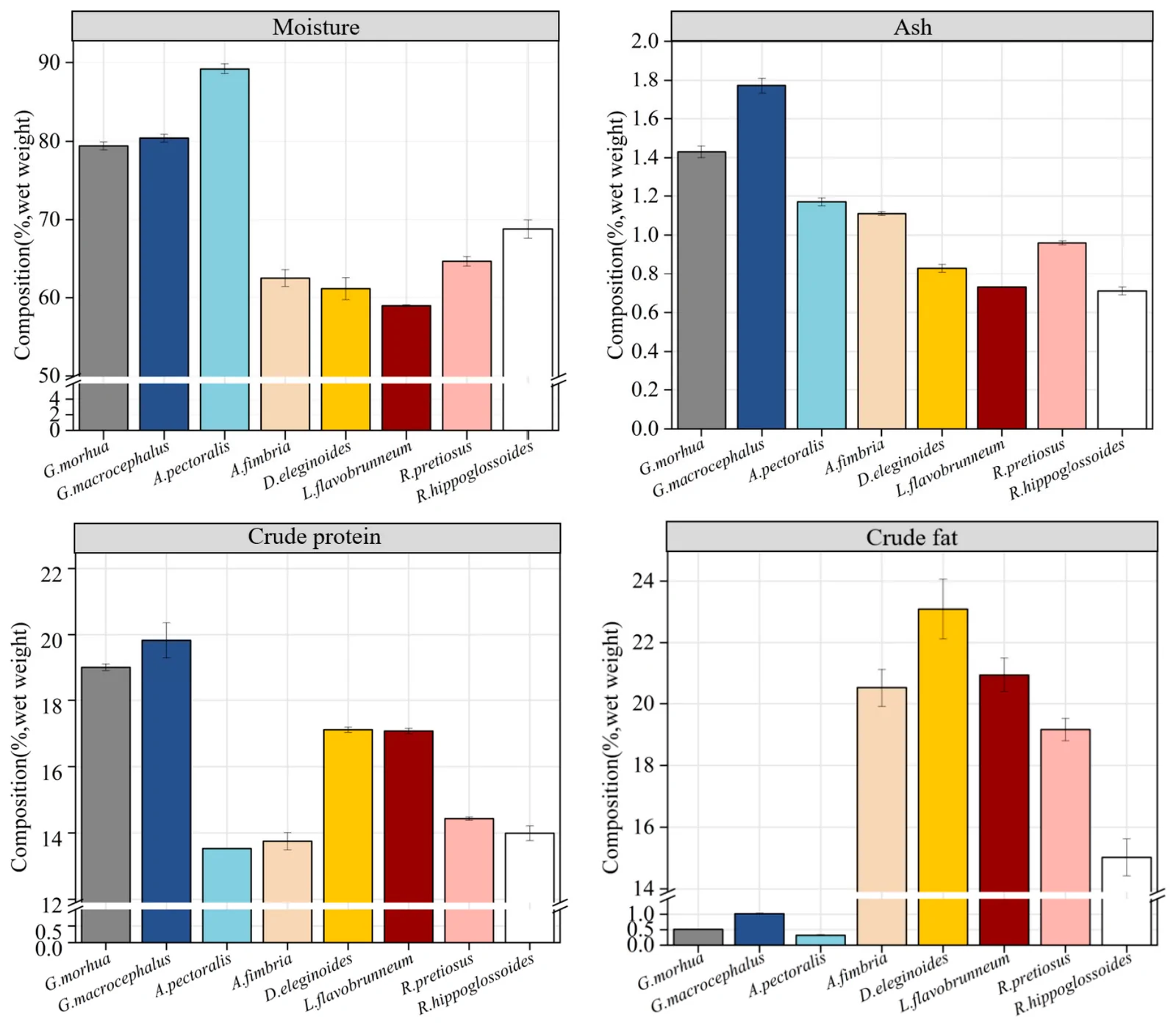
Proximate composition of eight species marketed as “cod”. Values are presented as mean ± SD of technical replicates from pooled species samples. The data should be interpreted descriptively.

The proximate composition of the eight authenticated species showed statistical variation in moisture, crude protein, crude lipid and ash content (Figure 2 and Table S3). A clear distinction was observed between Gadiformes and non-Gadiformes species, indicating that products marketed under the common commercial designation “cod” (鳕) might differ substantially in their basic nutritional composition.

The three Gadiformes species (*G. macrocephalus*, *G. morhua*, and *A. pectoralis*) exhibited relatively high moisture (79.42–89.25%) and ash (1.17–1.77%) content, coupled with very low crude lipid content (0.32–1.02%, wet-weight), consistent with the nutritional characteristics generally associated with lean whitefish [52]. According to the lipid classification proposed by Azrita et al. [53], all three Gadiformes species could be classified as very low-fat fish (<2% lipid). Among them, the two true cod species, *G. macrocephalus* (19.83 ± 0.53%) and *G. morhua* (19.01 ± 0.10%), contained relatively higher crude protein levels compared with *A. pectoralis* (13.53 ± 0.05%) and all authenticated non-Gadiformes species. Specifically, *G. macrocephalus* exhibited the highest protein content, which falls within the upper-intermediate part of the commonly reported range (15–25%) [54]. The relatively high protein proportion observed in the two *Gadus* species may be explained by their lipid storage strategy: Atlantic cod and Pacific cod are lean fish that store lipids predominantly in the liver rather than in muscle tissue, resulting in very low intramuscular lipid deposition and a correspondingly higher protein proportion on a wet-weight basis [55]. In addition, the protein-rich diet of these species is consistent with their high muscle protein content [56,57]. These results further support the long-standing perception of “cod” as high-protein, low-fat seafood [58].

In contrast, the five non-Gadiformes species (*A. fimbria*, *D. eleginoides*, *R. hippoglossoides*, *L. flavobrunneum*, *R. pretiosus*) exhibited different compositional characteristics. Moisture content decreased to 58.98–68.79%, while crude lipid content increased to 15.02–23.09%, classifying these species as high-fat fish (>8% lipid) [53], with *D. eleginoides* containing the highest lipid level (23.09 ± 0.97%). Crude protein content ranged from 13.75% to 17.11%, generally lower than that of true cod species. Although *D. eleginoides* and *L. flavobrunneum* met the criterion for high-protein fish (>17%), their substantially higher lipid content indicates a nutritional pattern very different from that expected for “cod” (鳕).

The contrasting proximate composition between Gadiformes and non-Gadiformes species primarily reflects species-specific physiological adaptations, including differences in lipid storage strategies and habitat. Gadiformes species generally store relatively little lipid in muscle tissue, whereas several non-Gadiformes species accumulate considerable intramuscular lipid, producing a richer texture but substantially altering the overall nutritional composition [21,23]. High-protein, low-fat foods like true cods are associated with cardiovascular benefits, including blood pressure reduction and decreased cardiovascular disease risk, aligning with contemporary dietary recommendations [59]. Consumers purchasing products labeled as “cod” (鳕) legitimately expect a lean, protein-rich white fish. Replacement with naturally high-fat species therefore changes the nutritional characteristics of the final product, particularly with respect to lipid intake and energy density.

Particular attention should be paid to *L. flavobrunneum* (escolar) and *R. pretiosus* (oilfish), both of which contained relatively high lipid levels (20.95% and 19.16%, respectively). Previous studies demonstrated that more than 90% of their lipids are composed of indigestible wax esters, excessive consumption of which has been associated with transient gastrointestinal symptoms [11,51]. Although the present study did not quantify wax esters directly, the molecular identification of these species highlights the importance of accurate species labeling to enable informed consumer choices.

### 3.3. Hydrolyzed Amino Acid Composition and Protein Nutritional Quality Assessment

#### 3.3.1. Hydrolyzed Amino Acid Composition

Seventeen hydrolyzed amino acids (HAAs) were detected in all eight authenticated species (Table 2). Glutamic acid (Glu: 2.04–2.82 g/100 g) and aspartic acid (Asp: 1.26–1.79 g/100 g) were the predominant amino acids across all species, accounting for a substantial proportion of total amino acids. These acidic amino acids are recognized as major contributors to umami taste in seafood [60]. Among essential amino acids (EAAs), lysine (Lys: 1.04–1.56 g/100 g) and leucine (Leu: 0.99–1.41 g/100 g) exhibited the highest abundance. *G. macrocephalus* and *G. morhua* exhibited relatively higher total HAA, essential amino acid (EAA), and conditionally essential amino acid (CEAA) contents compared with *A. pectoralis* and most non-Gadiformes species. However, some non-Gadiformes species, particularly *L. flavobrunneum* and *R. pretiosus*, also showed relatively high amino acid contents, with total HAA, EAA, and CEAA exceeding those of true cod species. Therefore, amino acid composition alone cannot reliably authenticate “cod” products, and molecular identification remains essential.

A notable species-specific difference was observed for histidine (His) content. *L. flavobrunneum* exhibited an exceptionally high His content (1.21 g/100 g), approximately threefold higher than that of the other examined species (0.25–0.45 g/100 g). This value exceeded previously reported levels in several histamine-rich fish species, including Spanish mackerel (*Scomberomorus niphonius*: 0.53 g/100 g) and yellowfin tuna (*Thunnus albacares*: 1.00 g/100 g), although it remained lower than bluefin tuna (*Thunnus thynnus*: 1.67 g/100 g) [61,62]. Histidine plays important physiological roles in hemoglobin synthesis, acid–base regulation, and immune function [63]. However, histidine-rich fish muscles may have an increased potential for histamine accumulation during inappropriate storage because microbial decarboxylation of histidine is the primary pathway for histamine formation [64]. Therefore, although the high His content of *L. flavobrunneum* contributes to its amino acid profile, strict and proper post-harvest handling is essential to minimize potential histamine-related risks. Notably, because *L. flavobrunneum* was represented by a single sample, this observation should be interpreted with caution.

The nutritional balance of fish protein was further evaluated based on EAA proportions. The EAA/TAA ratio of all eight species ranged from 37.89% to 40.25%, closely matching the FAO/WHO-recommended value (~40%), while the EAA/NEAA ratio ranged from 71.36% to 80.82%, exceeding the 60% threshold for high-quality protein sources [65,66]. Thus, all authenticated species possessed relatively balanced amino acid profiles, despite differences in taxonomy and lipid characteristics.

#### 3.3.2. Protein Nutritional Quality Evaluation

To further assess protein quality, the digestible indispensable amino acid reference ratio (DIAARR), digestible indispensable amino acid score (DIAAS), chemical score (CS; whole egg protein pattern), and essential amino acid index (EAAI) were calculated (Table 3). Because amino acid-specific digestibility data were not available, a single protein digestibility of “cod” (82%) was assumed for all species, and DIAARR and DIAAS values were shown as eDIAARR and eDIAAS using the FAO/WHO adult amino acid reference pattern [45,67].

The eDIAAS analysis revealed species-specific differences in the limiting amino acid. For *G. macrocephalus*, *G. morhua*, *D. eleginoides*, *L. flavobrunneum*, and *R. pretiosus*, valine (Val) was identified as the first limiting amino acid, whereas leucine (Leu) was the second limiting amino acid. In contrast, *A. pectoralis* and *R. hippoglossoides* had Val as the first limiting amino acid and His as the second, while *A. fimbria* showed His and Val as the first and second limiting amino acids, respectively. According to FAO/WHO recommendations, DIAAS values >100 indicate excellent protein quality, whereas values between 75 and 99 indicate good quality [45]. In this study, under the assumed uniform digestibility, *G. morhua*, *D. eleginoides*, *L. flavobrunneum*, *R. pretiosus*, and *R. hippoglossoides* showed eDIAAS values above 100, indicating that the estimated available amounts of indispensable amino acids met or exceeded the reference requirement. The remaining species showed eDIAAS values between 90 and 100, which remained in the good-quality range. These results demonstrate that, under the assumed digestibility, both Gadiformes species and non-Gadiformes species may provide dietary proteins with favorable indispensable amino acid profiles.

Using whole egg protein as the reference standard, sulfur-containing amino acids (SAA, Met + Cys) were the first limiting amino acids in all eight species. Nevertheless, lysine showed consistently high DIAARR and CS values (>1.00), suggesting that fish proteins are rich sources of lysine, which is nutritionally valuable because lysine is frequently the first limiting amino acid in cereal-based diets, and fish consumption may complement lysine deficiency in plant-derived proteins [67,68]. The EAAI values further confirmed high protein quality: all species exhibited EAAI values above 95, indicating well-balanced essential amino acid composition. Notably, the non-Gadiformes species *R. pretiosus* and *R. hippoglossoides* showed EAAI values >100, suggesting essential amino acid profiles comparable to or more balanced than whole egg protein. However, high EAAI values should not be interpreted as indicative of overall food quality, because lipid composition and potential undesirable components must also be considered.

### 3.4. Free Amino Acid Composition and Its Implications for Flavor Characteristics

Free amino acids (FAAs) are critical low-molecular-weight nitrogenous compounds that directly contribute to the sensory characteristics of seafood, particularly through their roles in umami, sweetness, and bitterness perception [69]. Seventeen FAAs were detected among the eight species (Table 4). Based on reported sensory properties, FAAs were classified into umami/sweet-tasting (Asp, Glu, Gly, Ala, Ser, Thr, and Pro) and bitter-tasting (Val, Met, Ile, Leu, Tyr, Phe, Trp, His, Lys, and Arg) groups.

Among Gadiformes, umami/sweet-tasting FAAs represented the predominant fraction of total FAAs. *G. morhua* and *G. macrocephalus* exhibited the highest proportions of umami/sweet-tasting FAAs (65.10% and 67.30%, respectively) and relatively high absolute concentrations (0.123 g/100 g and 0.104 g/100 g). This provides a potential biochemical explanation for the favorable sensory perception of true cod. Although *A. pectoralis* also showed a relatively high proportion of umami/sweet FAAs (51.13%), its absolute concentration was much lower (0.015 g/100 g), which may indicate a lower abundance of taste-active compounds relative to the true cod species.

In contrast, all five non-Gadiformes species displayed a higher proportion of bitter-tasting FAAs than umami/sweet-tasting FAAs. The most remarkable difference was observed in *L. flavobrunneum*, where bitter FAAs accounted for 94.88% of total FAAs and total FAA concentration was 0.697 g/100 g, approximately 3.7–24 times higher than other species. This was mainly attributed to an exceptionally high histidine content (0.616 g/100 g), representing approximately 88% of total FAAs. Such an unusual profile may contribute to the taste characteristics of escolar [70]. *R. pretiosus* also exhibited a relatively high proportion of bitter FAAs (69.91%), mainly due to elevated Lys, Val, and Leu contents. Although nutritionally important, excessive accumulation of these amino acids may be associated with bitter taste perception, negatively affecting sensory acceptance and creating a mismatch between consumer expectations and the actual flavor characteristics of products marketed as “cod” (鳕). These observations for *L. flavobrunneum* and *R. pretiosus* are based on single samples and require confirmation with larger sample sizes. In addition, FAA content reflects the potential taste-active composition of the samples; actual taste perception also depends on concentration thresholds, interactions among taste-active compounds, and the food matrix, and thus requires comprehensive validation in future studies.

### 3.5. Fatty Acid Composition and Nutritional Implications

Fatty acid composition is a key determinant of seafood nutritional value, especially omega-3 polyunsaturated fatty acids (ω-3 PUFA), including eicosapentaenoic acid (EPA, C20:5n-3) and docosahexaenoic acid (DHA, C22:6n-3) [71,72]. A total of 23 fatty acids were identified across the eight species, and the major fatty acids are presented in Table 5. The Gadiformes species exhibited a typical fatty acid profile of lean marine fish [71,73], characterized by relatively high PUFA proportions and abundant ω-3 PUFA. The dominant fatty acid distribution pattern was SFA > PUFA > MUFA, with total PUFA ranging from 30.50% to 39.22% of total fatty acids. EPA + DHA represented 29.94–37.10% of total fatty acids, with the highest proportions in *G. morhua* (37.10%) and *G. macrocephalus* (35.61%), substantially higher than many commonly consumed fish [74,75]. Within the lipid fraction, the ω-6/ω-3 PUFA ratios of all Gadiformes species were extremely low (0.03–0.08), indicating a predominance of anti-inflammatory ω-3 PUFA over ω-6 PUFA, which was considered a desirable dietary fatty acid balance [76,77].

Non-Gadiformes species exhibited fundamentally different fatty acid composition patterns, generally showing MUFA predominance (MUFA > SFA > PUFA or MUFA > PUFA > SFA), mainly contributed by oleic acid (C18:1n-9). The highest MUFA proportions were observed in *L. flavobrunneum* (79.24%) and *R. pretiosus* (69.33%). Their EPA + DHA proportions ranged from 5.81% to 14.09%, substantially lower than true cod species. Specifically, *L. flavobrunneum* exhibited the lowest EPA + DHA proportion (5.81%) among all species, approximately one-sixth of that of *G. morhua*. It should be noted that the fatty acid profiles of *L. flavobrunneum* and *R. pretiosus* are based on single samples; however, their high MUFA and low ω-3 PUFA patterns are consistent with previous reports for these species [11,51]. Because wax esters constitute more than 90% of the total lipid in these two species [11,70], a substantial proportion of the measured fatty acids may additionally not be bioavailable.

Although the fatty acid analytical method employed in this study provides proportions rather than absolute contents, these compositional data remain informative. Consumers purchasing “cod” products generally expect a lean fish with a lipid fraction dominated by PUFA, particularly ω-3 PUFA. However, several non-Gadiformes species examined here displayed distinct lipid profiles characterized by higher proportions of MUFA and lower proportions of EPA + DHA. In addition, the non-Gadiformes species had total lipid contents of 15.02–23.09% and SFA + MUFA proportions of 77.19–88.39%, thereby altering the energy density and lipid composition of the product relative to this expectation. Therefore, species misidentification or inaccurate labeling (without a scientific name) may affect not only taxonomic authenticity but also the lipid-related attributes of the product as perceived by consumers.

## 4. Conclusions

This study combined DNA barcoding with comprehensive nutritional and flavor-related profiling to characterize “cod” (鳕) products from two major Chinese e-commerce platforms. Among the 36 sampled products, eight fish species representing multiple orders were authenticated. Notably, 44.4% were non-Gadiformes species, demonstrating a substantial discrepancy between the commercial use of “cod” (鳕) and strict taxonomic identity. True cod species (*Gadus* spp.) exhibited relatively high protein quality, low lipid content, favorable essential amino acid profiles, high proportions of EPA + DHA, and a predominance of umami/sweet-tasting free amino acids—characteristics that align with consumer expectations for premium cod products. In contrast, non-Gadiformes exhibited relatively higher lipid content, substantially lower EPA + DHA levels, and, in some cases, a predominance of bitter-tasting free amino acids. Two products identified as *L. flavobrunneum* and *R. pretiosus* raise additional health concerns because of their known wax ester content, although this was not directly quantified in the present study.

Several limitations should be acknowledged. The sample size (n = 36) and the exclusive focus on two e-commerce platforms limit generalizability to the broader Chinese seafood market. Moreover, we also acknowledge that the pooled-sample design precludes formal species-level statistical inference, and that the nutritional comparisons should be regarded as indicative of the sampled commercial products. Species represented by single samples (*L. flavobrunneum* and *R. pretiosus*) should be interpreted with caution, as their nutritional data are preliminary and may not reflect species-level variation. Future studies with larger sample sizes and broader market coverage are warranted to confirm and extend these findings.

In conclusion, among the sampled products, the use of the same commercial name for products with different scientific names in Chinese e-commerce “cod” (鳕) products is not merely a taxonomic or labeling issue but can also result in substantial differences in nutritional composition and flavor characteristics. The integration of DNA-based species authentication with nutritional profiling offers an evidence-based framework for improving seafood labeling transparency, protecting consumer interests, and ensuring that the nutritional characteristics associated with authentic cod are accurately represented in the Chinese e-commerce market.

## Figures and Tables

**Table 1:** DNA barcoding identification results of 36 “cod” products from Chinese e-commerce platforms.

Sample ID	Product Label (as Purchased)	Species Identified by DNA Barcoding	Gadiformes Proportion
X1, X3, X5, X27	Silver cod	*Anoplopoma fimbria* (Sablefish)	0/11
X2, X4, X6, X7, X28, X29, X33		*Dissostichus eleginoides* (Patagonian toothfish)	
X8, X11, X16, X17, X18, X24, X31, X32, X34, X36	Various cod labels (e.g., “premium cod”, “cod steak”, “cod fillet”)	*Albatrossia pectoralis* (Giant grenadier)	13/16
X10, X20, X23		*Reinhardtius hippoglossoides* (Greenland halibut)	
X12, X19, X30		*Gadus macrocephalus* (Pacific cod)	
X21, X25	Pacific cod	*Gadus macrocephalus* (Pacific cod)	2/2
X13, X14, X15, X22, X35	Atlantic cod	*Gadus morhua* (Atlantic cod)	5/5
X26	Cod pieces	*Ruvettus pretiosus* (Oilfish)	0/1
X9	White cod	*Lepidocybium flavobrunneum* (Escolar)	0/1

Note: “Gadiformes proportion” indicates the number of products identified by DNA barcoding as Gadiformes species divided by the total number of products in that group with the same product label.

**Table 2:** Hydrolyzed amino acid composition of eight “cod” species (g/100 g, wet weight).

Amino Acid	*G. morhua*	*G. macrocephalus*	*A. pectoralis*	*A. fimbria*	*D. eleginoides*	*L. flavbrunneum*	*R. pretiosus*	*R. hippoglosoides*
Asp	1.79 ± 0.02	1.73 ± 0.01	1.26 ± 0.01	1.31 ± 0.03	1.64 ± 0.01	1.68 ± 0.01	1.73 ± 0.01	1.55 ± 0.01
Thr #	0.79 ± 0.05	0.77 ± 0.00	0.57 ± 0.02	0.61 ± 0.01	0.76 ± 0.01	0.84 ± 0.01	0.81 ± 0.01	0.74 ± 0.01
Ser	0.82 ± 0.01	0.81 ± 0.01	0.59 ± 0.04	0.59 ± 0.05	0.75 ± 0.01	0.72 ± 0.01	0.73 ± 0.01	0.68 ± 0.01
Glu	2.82 ± 0.01	2.78 ± 0.01	2.01 ± 0.02	2.10 ± 0.30	2.61 ± 0.01	2.72 ± 0.01	2.72 ± 0.03	2.61 ± 0.01
Gly	0.88 ± 0.01	1.04 ± 0.01	0.69 ± 0.04	0.73 ± 0.03	0.90 ± 0.01	0.80 ± 0.01	0.78 ± 0.01	0.66 ± 0.01
Ala	1.06 ± 0.01	1.06 ± 0.00	0.70 ± 0.01	0.77 ± 0.02	0.96 ± 0.01	1.03 ± 0.03	1.01 ± 0.04	0.87 ± 0.01
Cys	0.06 ± 0.02	0.06 ± 0.01	0.04 ± 0.01	ND	ND	ND	0.04 ± 0.01	0.03 ± 0.01
Val #	0.82 ± 0.01	0.80 ± 0.10	0.55 ± 0.05	0.60 ± 0.06	0.78 ± 0.01	0.87 ± 0.05	0.81 ± 0.01	0.73 ± 0.02
Met #	0.52 ± 0.01	0.52 ± 0.02	0.38 ± 0.05	0.42 ± 0.01	0.56 ± 0.01	0.56 ± 0.01	0.51 ± 0.03	0.47 ± 0.03
Ile #	0.74 ± 0.01	0.72 ± 0.01	0.53 ± 0.03	0.57 ± 0.03	0.71 ± 0.01	0.80 ± 0.01	0.78 ± 0.01	0.70 ± 0.02
Leu #	1.41 ± 0.00	1.34 ± 0.00	0.99 ± 0.10	1.03 ± 0.05	1.30 ± 0.01	1.40 ± 0.01	1.41 ± 0.02	1.31 ± 0.01
Tyr	0.66 ± 0.01	0.67 ± 0.04	0.44 ± 0.01	0.47 ± 0.01	0.61 ± 0.01	0.70 ± 0.01	0.64 ± 0.03	0.59 ± 0.01
Phe #	0.66 ± 0.01	0.65 ± 0.02	0.48 ± 0.03	0.52 ± 0.02	0.62 ± 0.02	0.68 ± 0.03	0.64 ± 0.02	0.57 ± 0.01
Lys #	1.52 ± 0.01	1.47 ± 0.02	1.04 ± 0.04	1.14 ± 0.01	1.42 ± 0.03	1.52 ± 0.03	1.57 ± 0.01	1.40 ± 0.02
His *	0.37 ± 0.00	0.36 ± 0.01	0.25 ± 0.05	0.27 ± 0.01	0.34 ± 0.01	1.21 ± 0.02	0.46 ± 0.01	0.32 ± 0.02
Arg *	1.12 ± 0.01	1.13 ± 0.01	0.83 ± 0.03	0.84 ± 0.01	1.05 ± 0.01	1.07 ± 0.08	1.07 ± 0.01	0.99 ± 0.03
Pro	0.58 ± 0.01	0.64 ± 0.03	0.42 ± 0.02	0.46 ± 0.01	0.59 ± 0.01	0.59 ± 0.01	0.54 ± 0.01	0.48 ± 0.02
∑TAA	16.63 ± 0.03	16.55 ± 0.04	11.77 ± 0.50	12.45 ± 0.56	15.58 ± 0.03	17.21 ± 0.22	16.24 ± 0.16	14.70 ± 0.05
∑EAA	6.46 ± 0.02	6.27 ± 0.05	4.54 ± 0.32	4.90 ± 0.14	6.14 ± 0.05	6.67 ± 0.10	6.52 ± 0.07	5.92 ± 0.01
∑NEAA	8.68 ± 0.02	8.79 ± 0.03	6.15 ± 0.10	6.44 ± 0.42	8.05 ± 0.42	8.26 ± 0.03	8.19 ± 0.09	7.47 ± 0.02
∑CEAA	1.49 ± 0.01	1.49 ± 0.01	1.08 ± 0.08	1.12 ± 0.01	1.40 ± 0.01	2.28 ± 0.10	1.53 ± 0.01	1.31 ± 0.05
(∑EAA/∑TAA)/%	38.86 ± 0.05	37.89 ± 0.23	38.54 ± 1.08	39.36 ± 0.68	39.40 ± 0.24	38.77 ± 0.12	40.17 ± 0.10	40.25 ± 0.07
(∑CEAA/∑TAA)/%	8.96 ± 0.08	9.00 ± 0.05	9.17 ± 0.29	8.98 ± 0.41	8.96 ± 0.02	13.26 ± 0.43	9.40 ± 0.05	8.91 ± 0.31
(∑EAA/∑NEAA)/%	74.45 ± 0.08	71.36 ± 0.78	73.78 ± 4.01	76.25 ± 2.88	76.31 ± 0.85	80.82 ± 1.07	79.65 ± 0.35	79.17 ± 0.24

Note: ND: no detection. # indicates essential amino acids for the human body; * indicates conditionally essential amino acids. ∑TAA: total amino acids; ∑EAA: total essential amino acids; ∑CEAA: total conditionally essential amino acids; ∑NEAA: total non-essential amino acids. Values are presented as mean ± SD of technical replicates from pooled species samples. The data should be interpreted descriptively.

**Table 3:** Estimated digestible indispensable amino acid reference ratio (eDIAARR), estimated digestible indispensable amino acid score (eDIAAS), chemical score (CS) and essential amino acid index (EAAI) of eight marketed “cod” (鳕) species.

	Species	Thr	Val	Ile	Leu	Lys	Met + Cys	Phe + Tyr	His	eDIAAS	EAAI
eDIAARR (FAO/WHO pattern)	*G. morhua*	1.56	1.01 *	1.22	1.14 **	1.56	1.24	1.59	1.14	101.15	
	*G. macrocephalus*	1.53	0.99 *	1.19	1.09 **	1.52	1.25	1.60	1.11	99.10	
	*A. pectoralis*	1.59	0.96 *	1.23	1.13	1.51	1.24	1.56	1.09 **	95.63	
	*A. fimbria*	1.61	0.99 **	1.25	1.11	1.55	1.21	1.56	0.93 *	92.71	
	*D. eleginoides*	1.60	1.04 *	1.23	1.11 **	1.56	1.26	1.56	1.12	103.81	
	*L. flavobrunneum*	1.58	1.03 *	1.27	1.10 **	1.50	1.18	1.61	3.59	103.28	
	*R. pretiosus*	1.65	1.03 *	1.31	1.16 **	1.63	1.20	1.57	1.41	103.01	
	*R. hippoglossoides*	1.65	1.02 *	1.30	1.19	1.63	1.21	1.58	1.12 **	101.95	
CS (Whole egg pattern)	*G. morhua*	1.06	0.91 **	0.91 **	1.05	1.39	0.74 *	0.92			98.07
	*G. macrocephalus*	1.03	0.90	0.89 **	1.00	1.35	0.75 *	0.93			96.23
	*A. pectoralis*	1.07	0.86 **	0.92	1.04	1.34	0.74 *	0.91			96.73
	*A. fimbria*	1.09	0.89 **	0.94	1.02	1.38	0.72 *	0.91			97.60
	*D. eleginoides*	1.08	0.94	0.92	1.02	1.38	0.75 *	0.91 **			98.40
	*L. flavobrunneum*	1.07	0.93 **	0.95	1.01	1.33	0.70 *	0.94			97.41
	*R. pretiosus*	1.12	0.93	0.98	1.07	1.45	0.72 *	0.91 **			100.34
	*R. hippoglossoides*	1.12	0.92 **	0.97	1.09	1.44	0.72 *	0.92 **			100.78

Note: * indicates the first limiting amino acid; ** indicates the second limiting amino acid.

**Table 4:** Free amino acid composition of eight “cod” (鳕) species (g/100 g, wet weight) and taste contribution.

Amino Acid	*G. morhua*	*G. macrocephalus*	*A. pectoralis*	*A. fimbria*	*D. eleginoides*	*L. flavobrunneum*	*R. pretiosus*	*R. hippoglossoides*
Umami/sweet								
Asp	0.004 ± 0.001	0.003 ± 0.000	0.001 ± 0.000	0.001 ± 0.001	ND	0.001 ± 0.000	ND	0.003 ± 0.000
Thr	0.016 ± 0.001	0.007 ± 0.001	0.001 ± 0.000	0.003 ± 0.001	0.002 ± 0.000	0.004 ± 0.000	0.005 ± 0.000	0.009 ± 0.000
Ser	0.023 ± 0.001	0.006 ± 0.000	0.003 ± 0.000	0.004 ± 0.000	0.002 ± 0.000	0.003 ± 0.000	0.003 ± 0.000	0.004 ± 0.000
Glu	0.015 ± 0.001	0.010 ± 0.001	0.003 ± 0.000	0.005 ± 0.000	0.001 ± 0.000	0.005 ± 0.000	0.001 ± 0.000	0.003 ± 0.000
Gly	0.025 ± 0.000	0.034 ± 0.002	0.003 ± 0.000	0.008 ± 0.000	0.003 ± 0.000	0.005 ± 0.000	0.012 ± 0.001	0.005 ± 0.000
Ala	0.027 ± 0.000	0.033 ± 0.000	0.003 ± 0.000	0.016 ± 0.000	0.008 ± 0.000	0.014 ± 0.000	0.014 ± 0.001	0.009 ± 0.000
Pro	0.013 ± 0.000	0.011 ± 0.001	0.001 ± 0.000	0.002 ± 0.000	0.001 ± 0.000	0.004 ± 0.000	0.004 ± 0.000	0.014 ± 0.001
Total umami/sweet	0.123 ± 0.002	0.104 ± 0.003	0.015 ± 0.001	0.039 ± 0.000	0.017 ± 0.000	0.036 ± 0.000	0.038 ± 0.000	0.046 ± 0.000
Bitter								
Val	0.008 ± 0.001	0.006 ± 0.001	0.002 ± 0.000	0.005 ± 0.000	0.002 ± 0.000	0.008 ± 0.001	0.006 ± 0.000	0.005 ± 0.000
Met	0.004 ± 0.000	0.004 ± 0.000	0.001 ± 0.000	0.003 ± 0.001	0.001 ± 0.000	0.007 ± 0.000	0.006 ± 0.000	0.003 ± 0.000
Ile	0.004 ± 0.000	0.003 ± 0.000	0.001 ± 0.000	0.003 ± 0.000	0.001 ± 0.000	0.003 ± 0.000	0.003 ± 0.000	0.002 ± 0.001
Leu	0.008 ± 0.001	0.005 ± 0.001	0.001 ± 0.000	0.004 ± 0.000	0.002 ± 0.000	0.005 ± 0.000	0.005 ± 0.000	0.004 ± 0.000
Tyr	0.005 ± 0.001	0.003 ± 0.000	0.001 ± 0.000	0.004 ± 0.000	0.001 ± 0.000	0.003 ± 0.000	0.003 ± 0.000	0.002 ± 0.000
Phe	0.004 ± 0.001	0.002 ± 0.000	0.001 ± 0.000	0.002 ± 0.000	0.001 ± 0.000	0.001 ± 0.000	0.002 ± 0.000	0.001 ± 0.000
Trp	0.008 ± 0.000	0.008 ± 0.001	0.005 ± 0.000	0.010 ± 0.001	0.006 ± 0.000	0.003 ± 0.000	0.008 ± 0.000	0.020 ± 0.001
Lys	0.018 ± 0.002	0.016 ± 0.000	0.001 ± 0.000	0.015 ± 0.000	0.006 ± 0.000	0.013 ± 0.001	0.053 ± 0.001	0.021 ± 0.000
His	0.003 ± 0.000	ND	0.000 ± 0.000	0.002 ± 0.000	0.002 ± 0.000	0.616 ± 0.002	0.003 ± 0.000	0.001 ± 0.000
Arg	0.004 ± 0.000	0.004 ± 0.001	0.001 ± 0.000	0.003 ± 0.000	0.001 ± 0.000	0.003 ± 0.001	ND	0.002 ± 0.000
Total bitter	0.064 ± 0.002	0.050 ± 0.002	0.014 ± 0.001	0.052 ± 0.000	0.023 ± 0.000	0.661 ± 0.002	0.088 ± 0.001	0.062 ± 0.001
Total FAA	0.187 ± 0.004	0.154 ± 0.005	0.029 ± 0.001	0.091 ± 0.001	0.040 ± 0.001	0.697 ± 0.002	0.126 ± 0.001	0.108 ± 0.001
Umami/sweet (%)	65.51 ± 0.368	67.30 ± 0.077	51.13 ± 0.979	42.92 ± 0.109	42.47 ± 0.229	5.12 ± 0.025	30.08 ± 0.064	42.88 ± 0.096
Bitter (%)	34.49 ± 0.368	32.70 ± 0.077	48.87 ± 0.979	57.08 ± 0.109	57.53 ± 0.229	94.88 ± 0.025	69.91 ± 0.064	57.12 ± 0.096

Note: ND: no detection. Values are presented as mean ± SD of technical replicates from pooled species samples. The data should be interpreted descriptively.

**Table 5:** Fatty acid composition of eight “cod” species (% of total fatty acids, mean ± SD).

Fatty Acid	*G. morhua*	*G. macrocephalus*	*A. pectoralis*	*A. fimbria*	*D. eleginoides*	*L. flavobrunneum*	*R. pretiosus*	*R. hippoglossoides*
C14:0	0.99 ± 0.15	0.97 ± 0.09	0.73 ± 0.01	5.60 ± 0.19	3.64 ± 0.07	ND	0.28 ± 0.01	5.18 ± 0.24
C16:0	15.59 ± 0.87	14.05 ± 0.89	13.74 ± 0.53	17.77 ± 0.52	14.75 ± 0.28	2.82 ± 0.21	5.06 ± 0.04	17.77 ± 0.50
C18:0	4.62 ± 0.66	4.72 ± 0.60	5.04 ± 0.05	2.82 ± 0.06	3.73 ± 0.09	1.02 ± 0.10	2.56 ± 0.14	4.23 ± 0.62
C16:1	1.43 ± 0.07	1.30 ± 0.07	1.23 ± 0.12	8.34 ± 0.53	7.90 ± 0.32	0.96 ± 0.20	1.70 ± 0.81	12.01 ± 0.40
C17:1	ND	ND	ND	0.74 ± 0.03	0.70 ± 0.18	19.33 ± 1.23	0.41 ± 0.13	ND
C18:1n-9	8.69 ± 0.12	9.78 ± 0.42	7.39 ± 0.37	23.67 ± 1.07	35.45 ± 0.81	46.35 ± 0.60	53.34 ± 1.27	23.30 ± 0.97
C20:1	1.81 ± 0.11	ND	2.12 ± 0.18	10.58 ± 0.56	15.89 ± 0.41	11.19 ± 1.99	10.61 ± 0.76	8.67 ± 1.43
C24:1	2.79 ± 1.03	4.20 ± 1.00	4.59 ± 1.30	0.51 ± 0.15	1.28 ± 1.25	0.92 ± 0.09	1.56 ± 0.60	1.45 ± 0.14
C20:4	1.74 ± 0.06	2.49 ± 0.37	0.83 ± 0.08	7.75 ± 0.51	0.37 ± 0.56	3.02 ± 0.16	1.22 ± 0.33	6.26 ± 0.96
C20:5 (EPA)	13.91 ± 0.54	13.37 ± 0.31	8.38 ± 0.02	8.18 ± 0.14	3.50 ± 0.09	0.90 ± 0.11	1.01 ± 1.56	7.22 ± 0.20
C22:6 (DHA)	23.19 ± 0.66	22.24 ± 0.84	21.57 ± 1.92	5.04 ± 0.31	6.11 ± 0.11	4.91 ± 0.40	10.65 ± 0.44	6.86 ± 0.86
∑SFA	46.20 ± 2.36	46.36 ± 2.40	54.56 ± 1.38	33.08 ± 0.35	27.37 ± 1.00	6.91 ± 0.43	12.61 ± 0.54	32.57 ± 0.98
∑MUFA	14.12 ± 1.09	15.28 ± 1.34	14.61 ± 0.93	44.11 ± 0.68	61.02 ± 0.32	79.24 ± 0.26	69.33 ± 2.04	45.42 ± 2.47
∑PUFA	39.22 ± 1.55	38.36 ± 1.40	30.50 ± 2.14	22.89 ± 0.68	11.49 ± 0.47	13.83 ± 0.18	17.79 ± 1.91	21.23 ± 2.13
∑ω-3PUFA	37.10 ± 1.19	35.61 ± 1.06	29.94 ± 1.90	13.21 ± 0.24	9.62 ± 0.14	10.28 ± 0.54	15.09 ± 1.76	14.09 ± 0.84
∑ω-6PUFA	2.12 ± 0.38	2.75 ± 0.60	0.83 ± 0.08	8.68 ± 0.47	1.58 ± 0.57	3.55 ± 0.64	2.70 ± 0.19	7.15 ± 1.35
EPA + DHA	37.10 ± 1.19	35.61 ± 1.06	29.94 ± 1.90	13.21 ± 0.24	9.62 ± 0.14	5.81 ± 0.45	11.66 ± 1.92	14.09 ± 0.84
ω-6/ω-3 ratio	0.06	0.08	0.03	0.66	0.16	0.35	0.18	0.51

Note: ND: no detection. Values are presented as mean ± SD of technical replicates from pooled species samples. The data should be interpreted descriptively.

## Data Availability

The original contributions presented in this study are included in the article/Supplementary Materials. Further inquiries can be directed to the corresponding author.

## Supplementary Materials

The following supporting information can be downloaded at: https://www.mdpi.com/article/10.3390/foods15193575/s1, Table S1: Species, GenBank accession number and citation information of the reference sequences used in this study; Table S2: GenBank accession numbers of *COI* sequences generated in this study; Table S3: Proximate composition of eight species marketed as “cod” (鳕) (%, wet weight).
