# Supplementary material for: DNA Barcoding and Comparative Nutritional Profiling of “Cod” (鳕) Products from Chinese E-Commerce Platforms
Source: Foods. 2026 Oct 8;15(19):3575. doi: 10.3390/foods15193575 (PMC13664775; doi:10.3390/foods15193575)
Supplement: Supplementary file 1 [file foods-15-03575-s001.zip › foods-4565649-supplementary.pdf]

Supplementary Table S1. Species, GenBank accession number and citation information of the reference sequences used in this study.

Species	GenBank Accession No.	Citation
Albatrossia pectoralis	MW128472.1	[25]
Anoplopoma fimbria	FJ164284.1, JQ353976.1(unpublished), KU168729.1	[26–27]
Dissostichus eleginoides	JN640622.1, JN640625.1, EF609344.1, EU074418.1	[28–30]
Gadus macrocephalus	MK011275.1	[31]
Gadus morhua	OQ210142.1, OQ210148.1, OQ210129.1	[32]
Lepidocybium flavobrunneum	FJ605805.1	[33]
Reinhardtius hippoglossoides	KC015874.1, HM421730.1, MH032539.1	[34–36]
Ruvettus pretiosus	NC_022493.1	[37]

Table S2 GenBank accession numbers of *COI* sequences generated in this study

Sample ID	GenBank accession number
X1	PZ886443
X2	PZ886444
X3	PZ886445
X4	PZ886448
X5	PZ886447
X6	PZ886555
X7	PZ886449
X8	PZ886450
X9	PZ886557
X10	PZ886452
X11	PZ886453
X12	PZ886455
X13	PZ886457
X14	PZ886458
X15	PZ886460
X16	PZ886464
X17	PZ886465
X18	PZ886583
X19	PZ886482
X20	PZ888612
X21	PZ886582
X22	PZ886489
X23	PZ886490
X24	PZ886563

X25	PZ886569
X26	PZ886497
X27	PZ886498
X28	PZ886502
X29	PZ886503
X30	PZ886507
X31	PZ886551
X32	PZ886561
X33	PZ886509
X34	PZ886560
X35	PZ886510
X36	PZ886584

Table S3 Proximate composition of eight species marketed as “cod” (%, wet weight)

	Moisture	Ash	Crude protein	Crude lipid
G. morhua	79.42±0.51	1.43±0.03	19.01±0.10	0.51±0.01
G. macrocephalus	80.42±0.50	1.77±0.04	19.83±0.53	1.02±0.02
A. pectoralis	89.25±0.57	1.17±0.02	13.53±0.05	0.32±0.02
A. fimbria	62.51±1.08	1.11±0.01	13.75±0.26	20.52±0.61
D. eleginoides	61.16±1.40	0.83±0.02	17.11±0.08	23.09±0.97
L. flavobrunneum	58.98±0.10	0.73±0.01	17.07±0.08	20.95±0.55
R. pretiosus	64.66±0.60	0.96±0.01	14.43±0.05	19.16±0.36
R. hippoglossoides	68.79±1.17	0.71±0.02	13.99±0.22	15.02±0.60

Note: Values are presented as mean ± SD of technical replicates from pooled species samples, the data should be interpreted descriptively.
